# Frontal-Subcortical Protein Expression following Prenatal Exposure to Maternal Inflammation

**DOI:** 10.1371/journal.pone.0016638

**Published:** 2011-02-10

**Authors:** Michelle Y. Deng, Sylvia Lam, Urs Meyer, Joram Feldon, Qi Li, Ran Wei, Lawrence Luk, Siew Eng Chua, Pak Sham, Yu Wang, Grainne Mary McAlonan

**Affiliations:** 1 Department of Psychiatry, University of Hong Kong, Hong Kong, Special Administrative Region, People's Republic of China; 2 Laboratory and Behavioral Neurobiology, Swiss Federal Institute of Technology Zurich (ETH), Schwerzenbach, Switzerland; 3 Genome Research Centre, University of Hong Kong, Hong Kong, Special Administrative Region, People's Republic of China; 4 State Key Laboratory for Brain and Cognitive Sciences, University of Hong Kong, Hong Kong, Special Administrative Region, People's Republic of China; 5 Department of Pharmacology, University of Hong Kong, Hong Kong, Special Administrative Region, People's Republic of China; Massachusetts General Hospital and Harvard Medical School, United States of America

## Abstract

**Background:**

Maternal immune activation (MIA) during prenatal life is a risk factor for neurodevelopmental disorders including schizophrenia and autism. Such conditions are associated with alterations in fronto-subcortical circuits, but their molecular basis is far from clear.

**Methodology/Principal Findings:**

Using two-dimensional differential in-gel electrophoresis (2D-DIGE) and mass spectrometry, with targeted western blot analyses for confirmation, we investigated the impact of MIA on the prefrontal and striatal proteome from an established MIA mouse model generated in C57B6 mice, by administering the viral analogue PolyI:C or saline vehicle (control) intravenously on gestation day (GD) 9. In striatum, 11 proteins were up-regulated and 4 proteins were down-regulated in the PolyI:C mice, while 10 proteins were up-regulated and 7 proteins down-regulated in prefrontal cortex (PFC). These were proteins involved in the mitogen-activated protein kinase (MAPK) signaling pathway, oxidation and auto-immune targets, including dual specificity mitogen-activated protein kinase kinase 1 (MEK), eukaryotic initiation factor (eIF) 4A-II, creatine kinase (CK)-B, L-lactate dehydrogenase (LDH)-B, WD repeat-containing protein and NADH dehydrogenase in the striatum; and guanine nucleotide-binding protein (G-protein), 14-3-3 protein, alpha-enolase, olfactory maker protein and heat shock proteins (HSP) 60, and 90-beta in the PFC.

**Conclusions/Significance:**

This data fits with emerging evidence for disruption of critical converging intracellular pathways involving MAPK pathways in neurodevelopmental conditions and it shows considerable overlap with protein pathways identified by genetic modeling and clinical post-mortem studies. This has implications for understanding causality and may offer potential biomarkers and novel treatment targets for neurodevelopmental conditions.

## Introduction

Dysfunction of frontal-subcortical circuits is implicated in psychiatric conditions with developmental origin, including schizophrenia [1] and autism [2]. The prefrontal cortex (PFC) is intimately linked to striatum, pallidum and thalamus via a serial of frontal-subcortical circuits [3]. The PFC is responsible for executive function and is heavily involved in social-emotional processes [4]. Dysfunction of this region results in disorganization of thoughts and actions, part and parcel of the psychopathology of schizophrenia. The dopamine rich striatal region interfaces between the PFC and other brain regions. Therefore, disruption to prefrontal cortical networks incorporating the striatum are thought crucial to schizophrenia [5] and also implicated in autism [2], [6]. Consistent with this, fronto-striatal prefrontal circuit anomalies have been detected in autism [2], [6], [7] and first episode schizophrenia prior to medication [1], [8], [9]; and functional imaging and sensorimotor gating abnormalities in both conditions are consistent with fronto-striatal perturbation [6], [10], [11], [12]. However, the molecular basis of frontal-subcortical dysfunction is uncertain, therefore, novel treatment targets remain under-exploited.

Proteomics holds potential to undercover pathological mechanisms at the molecular level. Compared to conventional analysis, two-dimensional differential in-gel electrophoresis (2D-DIGE) has higher reproducibility and sensitivity because of its internal standard design which minimizes gel-to-gel variation, improves spot-matching, reduces number of gels needed, and permits quantitative analysis of small sample amounts [13], [14]. Recently, post-mortem proteomic profiles of different brain regions from patients with schizophrenia [15], [16], [17], [18], [19] have been investigated. The expression of proteins involved in cell communication, signal transduction, cellular metabolism, synaptic plasticity, cell growth and oxidation proteins differs in samples from patients who had schizophrenia. However drug treatment, chronicity and cause of death, may confound interpretation and such studies cannot easily probe causative mechanisms. In contrast, animal models allow direct examination of aetiological risk factors on brain proteome. For example, rats with acute methamphetamine-induced behavioral sensitization have changes in mitochondrial dysfunction, oxidative, cytoskeletal, and synaptic proteins, which may hold clues to the nature of drug-induced psychosis [20], [21]. In a schizophrenia model simulated by neonatal hippocampal lesions in rats, reduced protein expression in the PFC has been reported, with alterations in proteins involved in neurotransmitter signaling systems [22].

Maternal immune activation (MIA) during prenatal life increases risk of development of schizophrenia and related illnesses such as autism [23], [24], [25], [26]. A substantial body of work has validated MIA in rodents as a means to simulate neuroanatomical [27], [28], neurochemical [29], [30], behavioural [31] and gene expression [32] abnormalities similar to those in schizophrenia, autism and related disorders. In this study, we adopted an established mouse model of MIA to study the effects of prenatal immune challenge on protein expression in striatum and PFC using 2D-DIGE, coupled with western blot analysis of selected proteins. The MIA model is based on prenatal maternal treatment with polyriboinosinic- polyribocytidilic acid (PolyI:C). PolyI:C is a synthetic analogue of virus-specific double-stranded RNA, which induces a cytokine-associated acute phase response typically seen following viral infections. As reviewed elsewhere [33], [34], prenatal PolyI:C treatment in rodents induces a variety of behavioral, cognitive and pharmacological dysfunctions primarily implicated in schizophrenia and autism. Hence, the prenatal PolyI:C model is highly suitable for proteomic analyses because of its solid face validity to schizophrenia and related disorders.

## Results

### 2D-DIGE Processing and Protein Identification

**Figure 1:**
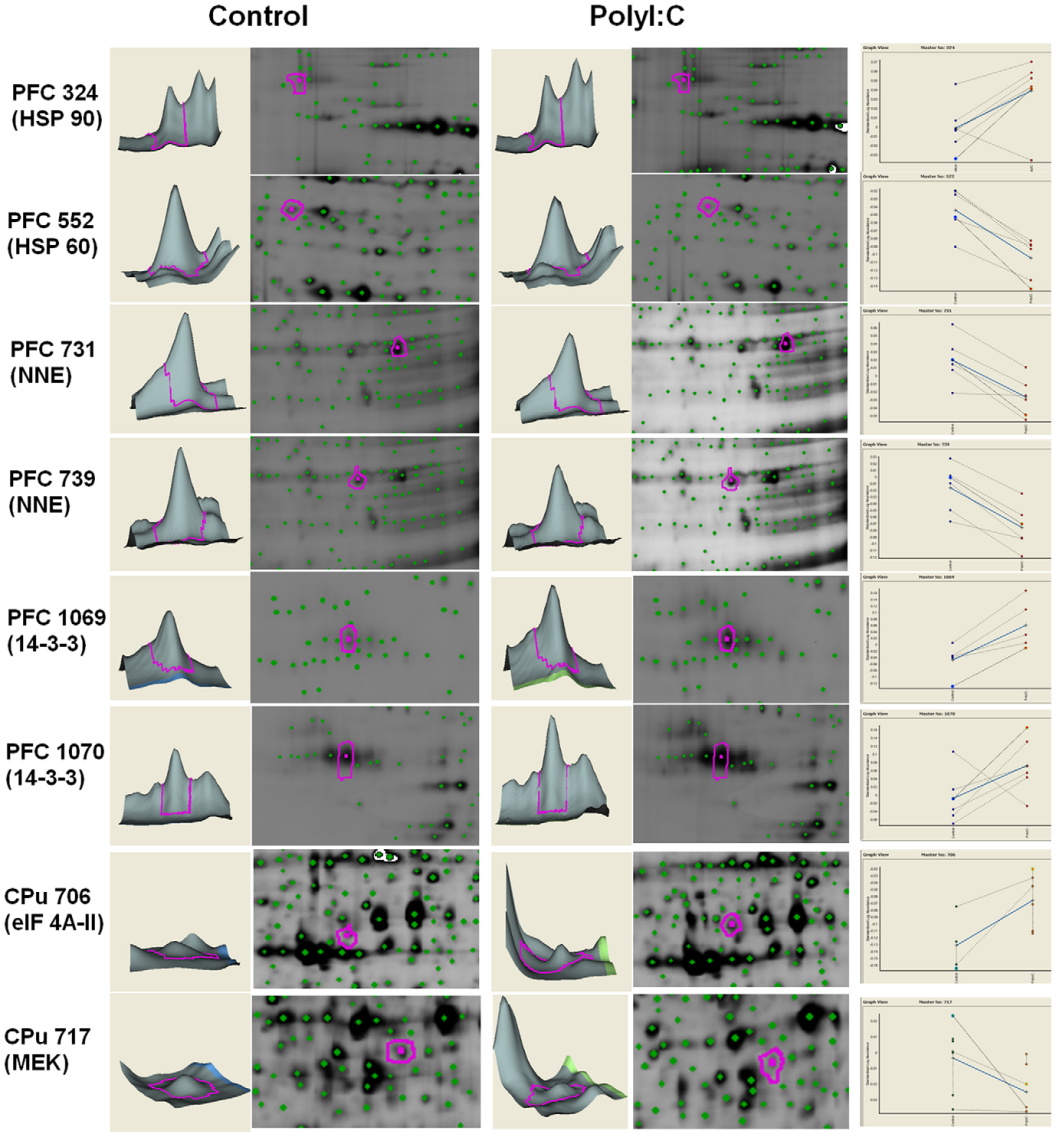
Differential expression patterns of proteins-of-interest and their abundances relative to the internal standard in the BVA module.

Total soluble brain proteins from CPu and PFC labeled with Cy3 or Cy5 fluorescent dyes, mixed to form random pairs on each 2D-DIGE gel, were evenly distributed across the pH 4–7 range and separated between 10 to 250 kDa on the 12% SDS-PAGE. For CPu, an average 1500 spots were detected in each image by DeCyder DIA module, and around 1300 spots included for further BVA module analysis after protein exclusion filters applied (Area<250; Max Peak<100; Max Volume<10,000; Max Slope>1.75). For PFC, around 1800 spots were detected and 1300 spots were included for BVA module with similar protein exclusion filters (Area<250; Max Peak<200; Max Volume<25,000; Max Slope>1.5). In BVA module, protein spots detected from each image were automatically matched to the master image which contained the most protein spots in an experiment and each protein's standardized abundance volume was calculated against the internal standard. The protein fold changes were of a similar order of magnitude to other studies which have adopted 2D-DIGE techniques [35]. In addition, protein spots were manually checked to exclude dust particles and other artifact before further statistical analysis. Independent Student *t*-tests compared protein expression between PolyI:C- treated mice and controls. According to the significance criteria above, 15 spots in CPu showed significant alteration in expression (p<0.05; presented >75% images) in PolyI:C-treated mice compared to the control group. Of these 15 spots, 11 spots showed up-regulation and 4 spots showed down-regulation. Table 1 lists the 9 proteins identified by mass spectrometry. In PFC, compared to control group, 18 spots with significant alteration in expression were detected (p<0.05; presented in >75% images) in PolyI:C-treated mice,of which 10 spots were detected as up-regulated and 8 spots down-regulated. Nine protein spots were identified using mass spectrometry (Table 2), including olfactory marker protein; alpha-enolase protein; Guanine nucleotide-binding protein (G-protein); protein kinase C inhibitor protein 1 (KCIP-1) and heat shock proteins. Figure 1 shows representative expression patterns.

**Table 1:** Differential protein expression in striatum of PolyI:C-treated mice compared to controls.

Classification	Spot No.	Fold change	*p* value	Protein name	Protein MW (kDa)	Protein PI	Protein score	C.I.%	Pep. count	NCBInr accession number
Isomerase	CPu356	1.06 down	0.02	WD repeat-containing protein 1(Actin-interacting protein 1) (AIP1)	67.0492	6.11	103	99.998	11	gi|12836360
	CPu357	1.09 down	0.023	WD repeat-containing protein 1(Actin-interacting protein 1) (AIP1)	67.0492	6.11	82	99.798	10	gi|12835959
Transferase	CPu717	1.05 down	0.049	Dual specificity mitogen-activated protein kinase kinase1 (MAP kinase kinase 1) (MAPKK 1 or MEK1)	43.7885	6.24	186	100	11	gi|7670399
	CPu664	1.12 up	0.0009	Creatine kinase B-type	42.9714	5.4	399	100	14	gi|10946574
	CPu650	1.09 up	0.047	Creatine kinase B-type	42.9714	5.4	142	100	8	gi|10946574
	CPu659	1.09 up	0.05	Creatine kinase B-type	42.9714	5.4	768	100	21	gi|10946574
Hydrolase/RNA binding protein	CPu706	1.16 up	0.036	Eukaryotic initiation factor4A-II	46.6009	5.33	194	100	10	gi|56605748
Oxido-reductase	CPu907	1.07 up	0.0045	L-lactate dehydrogenase B chain (LDH-B) (LDH-H)	36.8341	5.7	82	99.773	7	gi|6678674
	CPu1070	1.05 up	0.0024	NADH dehydrogenase (ubiquinone) iron-sulfur protein 3	30.3016	6.67	279	100	15	gi|20071222

**Table 2:** Differential protein expression in prefrontal cortex of PolyI:C-treated mice compared to controls.

Chaperone	PFC324	1.1 up	0.028	Heat shock protein HSP 90-beta	83.5712	4.97	466	100	29	gi|123681
	PFC522	1.15 down	0.003	Heat shock protein HSP60	60.8849	5.38	163	100	8	gi|51452
Lyase	PFC731	1.11 down	0.013	Alpha-enolase (2-phospho-D-glycerate hydro-lyase)	47.6345	6.63	288	100	17	gi|6679651
	PFC739	1.15 down	0.013	Alpha-enolase (2-phospho-D-glycerate hydro-lyase) (Non-neural enolase)	47.4533	6.37	118	100	11	gi|70794816
Hydrolase	PFC1061	1.07 down	0.029	Platelet-activating factor acetylhydrolase IB subunit	25.7242	5.57	196	100	4	gi|33440467
Signaling protein	PFC942	1.07 up	0.023	Guanine nucleotide-binding protein G(I)/G(S)/G(T) subunit beta-1	38.1513	5.6	470	100	13	gi|6680045
	PFC1069	1.29 up	0.028	14-3-3 protein zeta/delta (Protein kinase C inhibitor protein 1) (KCIP-1)	27.9248	4.73	104	99.999	8	gi|6756041
	PFC1070	1.2 up	0.059	14-3-3 protein zeta/delta (Protein kinase C inhibitor protein 1) (KCIP-1)	28.1829	4.77	183	100	12	gi|3065925
	PFC1176	2.95 up	0.038	Olfactory marker protein	18.8547	5	128	100	7	gi|6754936

### Multivariate Statistical Analysis of Full Proteome Profile

**Figure 2:**
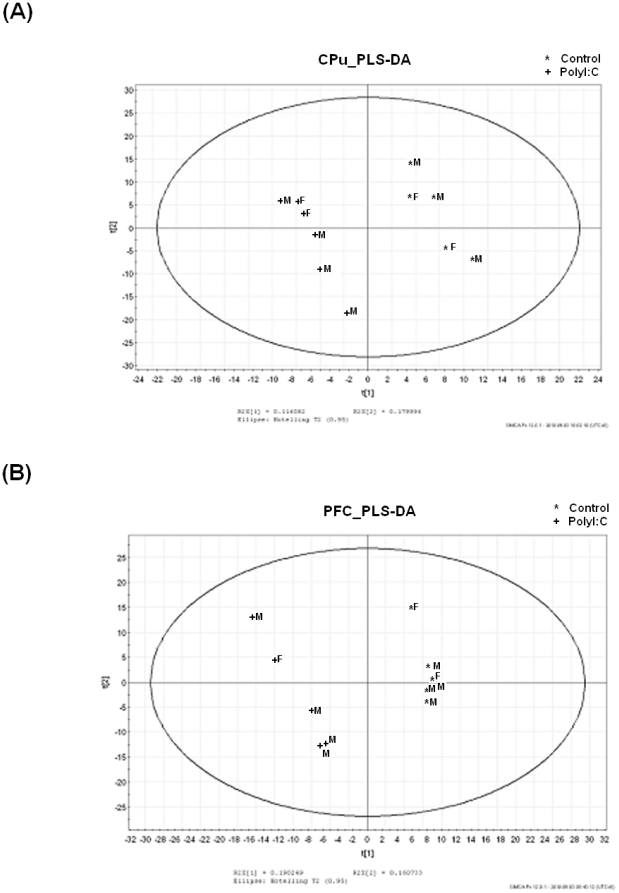
PolyI:C mice group was differentiated from controls using PLS-DA analysis of the whole CPu (A) and PFC (B) proteome. M, male; F, female.

In CPu (Figure 2, A) and PFC (Figure 2, B) respectively, 790 and 524 protein spots present in at least 11 out of 14 (>75%) images were included in partial least squares-discriminant analyses (PLS-DA). The PLS-DA showed a clear separation between groups for both regions (Figure 2).

### Western Blot Validation

**Figure 3:**
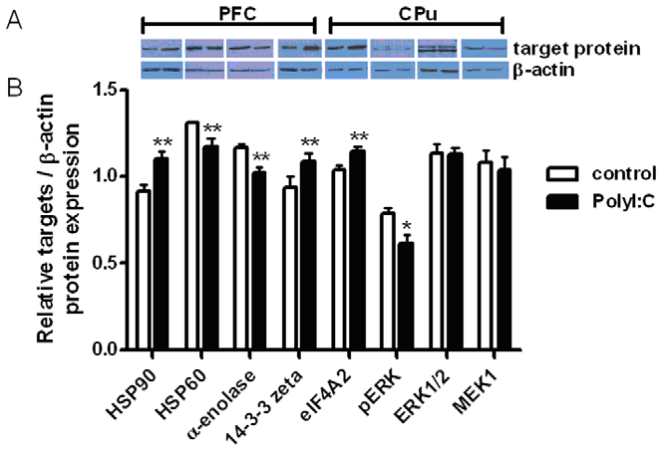
Western blot analysis of the differentially expressed target proteins in PolyI:C and control group. (A) Gel image of protein products in the control and PolyI:C group. (B) The relative expression of the target proteins/β-actin protein. Columns show mean ± SEM (n = 8 for PFC and n = 6 for CPu). *p<0.05 and **p<0.005 vs. corresponding control group.

Differentially expressed proteins detected by 2-D gel and mass spectrometry were confirmed using western blot analysis of selected proteins (Figure 3). In PFC, the expression of HSP-90 and 14-3-3 zeta were significantly up-regulated while HSP-60 and α-enolase were significantly down-regulated in the PolyI:C group as compared with the control group. In CPu, protein expression of eIF4A2 was significantly up-regulated. Down-regulation of MEK1 did not reach significance, but a significant down-regulation of pERK was consistent with lower MEK1 in the PolyI:C group compared with saline control.

## Discussion

Using an epidemiologically motivated neurodevelopmental animal model of schizophrenia and related disorders, we show for the first time that prenatal immune challenge in the form of a viral-like acute phase response triggered in early/middle gestation (GD9) in mice leads to long-lasting changes in protein expression at the proteome level. Our quantitative 2D-DIGE techniques revealed differential expression of proteins in the striatum and PFC of adult mice prenatally exposed to the viral mimic PolyI:C relative to adult control mice. More specifically, proteins involved in the MEK/ERK pathway, oxidation and auto-immune targets were found to have altered expression in PolyI:C exposed mice, including dual specificity mitogen-activated protein kinase kinase 1 (MEK1), eukaryotic initiation factor (eIF) 4A-II, creatine kinase (CK)-B, L-lactate dehydrogenase (LDH)-B, WD repeat-containing protein and NADH dehydrogenase in the CPu (Table 1); guanine nucleotide-binding protein (G-protein), 14-3-3 protein zeta/delta (KCIP-1), alpha-enolase, olfactory maker protein and heat shock protein (HSP)-60, and 90-beta in the PFC (Table 1). These proteome wide results were generally confirmed for 8 selected proteins using western blot (Figure 3) and PLS-DA multivariate analysis of the full proteome profile clearly separated PolyI:C and control groups (Figure 2).

In the maternal infection model, cytokine elevation rapidly triggers cellular defense programs [36]. Smith and Patterson concluded several cytokine signaling pathways, such as NF-κB and/or JAK/STAT, are involved in the modulation of cell proliferation, differentiation and migration during prenatal brain development. Activation of JAK/STAT pathway is particularly important in neuronal regeneration, embryonic development [37], neurogensis and gliogensis [38]. Pro-inflammatory cytokines have been shown to inhibit cortical neuron dendrite development in embryonic day 18 rat cortical cultures [39]. In our study, when triggered by pro-inflammatory cytokines, the intracellular signal transduction networks modified downstream of JAK/STAT, included the mitogen-activated protein kinase (MAPK) cascade which contains Ras/Raf, MEK, ERK. Confirmatory western blot analysis indicated lower level of phosphorylated ERK (pERK) was indeed a consequence of prenatal cytokine exposure, probably due to down-regulation of MEK. This finding is consistent with cell-culture study showing viral infection interferes with ERK1/2 phosphorylation in hippocampal neurones [40].

Post-mortem studies in patients of schizophrenia suggest the MAPK pathway is disrupted [41] with decreased MAPK1 gene expression reported in schizophrenia post-mortem samples [42]. In addition, preclinical investigation suggests the MAPK pathway is modified by anti-psychotic drug treatment [43], [44]. Chronic treatment with olanzapine activates the MAPK pathway leading to phosphorylation of MEK1/2 and ERK1/2 [43], suggesting that the lower MEK1 observed in our study may constitute a potential pharmacological target. Consistent with this, epigenetic regulation of MEK1 via methylation of an upstream CpG island has been reported to correlate with antipsychotic use in schizophrenia [44]. In addition, in both the knock-out mouse model of Fragile X and peripheral blood lymphocytes from individuals with fragile X, a condition associated with autism, Berry-Kravis *et al* [45] showed delayed phosphorylation of ERK, partly overlapping our observation that PolyI:C mice have lower MEK and pERK.

Activation of STAT3 trigged by cytokines may be rapidly inhibited by MEK-ERK negative regulation [46]. Our finding that prenatal inflammation down-regulates expression of MEK and pERK in the CPu region suggests the prenatal PolyI:C exposure might disrupt this feedback circuit, and have potentially widespread impact upon multiple cellular processes. The MAPK cascade is a vital cellular pathway for neuronal differentiation and function [47]. The 14-3-3 protein is key intracellular binding protein and one of its actions is to facilitate RAF activation of MEK [48]. Heat shock protein (HSP)-90 is also required to activate the RAF signaling cascade [49]. Our results showing both 14-3-3 and HSP-90 proteins were up-regulated in PolyI:C exposed offspring might indicate a facilitation of RAF to compensate for lower expression of MEK. Moreover, activation of HSP-90 appears to be coupled to the activation of IL6/STAT3 [50], and is over expressed in patients with schizophrenia [51], while 14-3-3 proteins were have been reported to be elevated in nucleus accumbens of isolation reared rats [52], and in the posterior hippocampus of post-mortem tissue from patients with schizophrenia [17].

Another important function of HSP90 is to ‘restrain’ activation of ErbB receptor netweork by ligands such as neuregulin 1 (*nrg*1). ERK phosphorylation is an end result of ErbB activation. In addition to possible disruption of MAPK signaling in schizophrenia and related conditions discussed above, *nrg*1 is a susceptibility gene for schizophrenia and expression of ErbB3 protein is defective in schizophrenia [53]. Our finding that a key inhibitory regulator of neuregulin-ErbB is up-regulated in the MIA model along with a down-regulation of MAPK signaling is therefore compelling, suggesting the same pathways are targeted by genetic and environmental perturbations relevant to neurodevelopmental conditions.

There is considerable cross-talk between MAPK pathway and others such as PI3K/PTEN, mTOR and fragile X mental retardation protein (FMRP) pathways [54]. PTEN mutations are associated with autism [55], [56], as are tuberose sclerosis and fragile X, which disrupt mTOR signaling. These pathways modulate protein translation via the eIF4 family proteins. In PolyI:C exposed offspring the eukaryotic initiation factor (eIF) 4A-II protein was up-regulated in the CPu. Recently novel association of the eIF4E protein component of this family with autism was reported [57]. Thus disruption of a number of critical converging pathways including MAPK, PTEN, mTOR and FMRP appears important in neurodevelopmental conditions, either as a consequence of genetic or environmental risk factors during prenatal life [58]. GSK3b interfaces between these pathways [59], [60], inhibited by MEK and mGLUR signaling [61] and increased by PTEN inhibition of PI3K/AKT. Lastly, in addition to facilitating Raf, protein 14-3-3, up-regulated in PolyI:C exposed mice regulates GSK3b interactions including phosphorylation of Tau [62]. These multiple clues pointing to GSK3b may be important to follow up in future studies because GSK3b inhibitors, such as Lithium, offer potential therapeutic options [61], [63].

Down-regulation of heat shock protein (HSP)-60 detected in PFC of PolyI:C exposed offspring, agrees with other schizophrenia-like models [52], [64]. In Roncada's study [52], isolation-reared rats showed significant pre-pulse inhibition (PPI) deficits correlated with the level of HSP-60 decrease. The MIA model has also reliably been shown to precipitate PPI impairment [28], [31]. HSP-60 is a proposed auto-immune target in schizophrenia [65], [66]. Similarly, alpha-enolase (NNE), significantly down-regulated in PolyI:C exposed offspring, is considered an auto-antigen [67]. In the MK801 rat model, NNE increased in the thalamus of MK801 short-term treated rats, but decreased with long-term treatment [64]. Combined with the evidence of lower HSP-60, which echoes reduction in liver proteome of patients with schizophrenia [65], our results raise the issue of whether the immune system is chronically affected following activation in fetal life. Certainly immune mechanisms have been suggested to explain glutamergic and dopaminergic disregulation characteristic of schizophrenia [68], and autism is accompanied by persistent immunological anomalies [69]. Proteomic and transcriptome studies in schizophrenia are also consistent with dysregulation of immune pathways [42], [70].

Other proteins altered in PolyI:C mice included creatine kinase (CK), actin-interacting proteins, L-lactate dehydrogenase proteins, NADH dehydrogenase iron-sulfur (Fe-S) proteins and WD repeat protein 1. The direction of change identified here is often, but not always, similar to that reported in analyses of schizophrenia post-mortem proteome. For example, NADH dehydrogenase was up-regulated here and has previously been reported as upregulated in schizophrenia posterior hippocampal post-mortem samples but reduced in anterior hippocampus [17]. CK type B was up-regulated here similar to findings in schizophrenia post-mortem tissue [16]. Moreover, CK isoforms are associated with the 14-3-3 protein complex [71], [72] so, that both are up regulated within this model is internally consistent. In contrast, WD repeat protein 1 has been reported as up-regulated in schizophrenia post-mortem tissue but was down-regulated in our model [70]. Possible reasons for divergent findings are many-fold, but may include contribution from medication exposure and illness progression in the clinical samples.

Our study has a number of limitations. A standard global scale proteomic analysis was applied to brain homogenate lysates. However, this approach does not fully sample different intracellular organelles in the brain. A further limitation was the modest rate of successful protein identification in mass spectrometric analysis, particularly in PFC. This false negative error may be because the regional homogenate was insensitive to low protein abundance. Given that the gels are loaded with a fixed protein quantity, more proteins separated, theoretically means lower concentrations of individual proteins. One solution is the use of subcellular fractions such as mitochondria, nuclear and plasma membrane which can further separate from brain lysates with chemical or physical fractionation techniques. Fractionation techniques, though challenging, will form good future partnership with proteomics [22].

In conclusion, prenatal immune challenge causes changes across the fronto-striatal proteome echoing findings from diverse studies on post-mortem tissue, genetic analyses and animal model systems. Specifically, our data fits with evidence for disruption of critical proteins within converging intracellular pathways involving MAPK in neurodevelopmental conditions. Further investigation of protein pathways in this and other related models may hold promise for further identification of disease biomarkers and novel treatment targets.

## Materials and Methods

### Animals and Sample Purification

C57BL6/J mice were used for the experiment. All procedures described in the present study had been previously approved by the Cantonal Veterinarian's Office of Zurich (permit number 28-2009) and are in agreement with the principles of laboratory animal care in the *Guide for the Care and Use of Laboratory Animals* (National Institutes of Health Publication No. 86-23, revised 1985). All efforts were made to minimize the number of animals used and their suffering. After local ethic review board approval, and following previously published protocol [27], [28], [31], 5mg/kg PolyI:C (potassium salt dissolved in saline, Sigma Aldrich) was administered in an injection volume of 5ml/kg to pregnant C57BL6/J mice on gestation day (GD) 9 via the tail vein. Control animals received 5ml/kg saline via tail vein on GD9. The resulting offspring were weaned and sexed at postnatal day (PND) 21. Littermates of the same sex were caged separately, three to four per cage. At adult age (PND 70), 7 mice (4 male and 3 female offspring) from each prenatal treatment group (saline and PolyI:C) were randomly selected from different litters. Hence, a total of 14 mice (7 control, 7 PolyI:C) were included in the 2D-DIGE analyses. Animals were sacrificed by decapitation and brains dissected, immediately frozen and stored at −80°C. Brain tissue from striatum (CPu) and PFC of saline- and PolyI:C-treated mice were individually thawed and homogenized in 2D-DIGE lysis buffer (30mM Tris, 7M Urea, 2M Thiourea, 4% CHAPS, pH 8.5; at 4°C) for 30 seconds, then centrifuged at 15,000×g for 15 min to remove cell debris. Supernatant was collected and quantified with 2-D Quant Kit (Amersham Biosciences) before loading gels for 2D-DIGE separation.

### 2D-DIGE Analysis and Protein Identification by Mass Spectrometry

2D-DIGE analyses of CPu and PFC samples were conducted independently. The procedure has been described previously [35]. Briefly, for each region, 50µg of protein was randomly labeled with 400 pmol of CyDye DIGE Fluor minimal dye (GE Healthcare), Cy3 or Cy5, and the pooled internal standard with Cy2. Labeled samples were randomly mixed and assigned to seven gels, each of which contained Cy2-, Cy3- and Cy5-labeled samples, respectively [73]. Mixed pre-labeled samples were loaded to the 18cm, pH 4–7, IPG DryStrips (Bio-Rad) for first dimensional isoelectric focusing (IEF) separation (Bio-Rad IEF system), and secondary dimensional separation by 12% SDS-PAGE (Bio-Rad vertical system). Fluorescent images were obtained by Typhoon™ 9410 scanner (GE Healthcare). For each region, a total of 14 images from 7 gels with good separation quality were analyzed using DeCyder software version 6.5 containing Differential In-gel Analysis (DIA) module and Biological Variation Analysis (BVA) module (Amersham Biosciences). The abundance of protein spots was intra- and inter- gel normalized according to the internal standard. Statistical analysis of different protein spot intensity between groups was performed using independent Student's *t*-tests run on the normalized protein abundance in DeCyder software. Protein spots with *p*-value less than 0.05 and present in at least 11 out of 14 (>75%) images [65] were considered significant. Spots of interest were picked from the silver-stained 2D-DIGE gels and followed by in-gel trypsin digestion and peptide extraction [35] for protein identification by tandem mass spectrometry analysis using ABI 4800 MALDI TOF/TOF™ MS Analyzer (Applied Biosystems, Foster City, CA, USA). The combined peptide mass fingerprinting (PMF) and MS/MS peptide fragmentation data were submitted to the NCBInr database and SwissProt database using the software GPS Explorer, version 3.6 (Applied Biosystems) and in-house MASCOT version 2.2 (Matrix Science) with parameters: one missed cleavage allowed; initial mass tolerance of 75ppm; fixed modification, carbamidomethyl (Cys); variable modification, oxidation (Met); precursor tolerance of ±0.2 Da. For all significant protein identifications, both protein and total ion scores were above or equal to C.I. 95% [35].

### Multivariate Statistical Analysis of Full Proteome Profile

Multivariate analysis using partial least squares-discriminant analysis (PLS-DA) was performed to examine whether PolyI:C groups could be separated from control groups on the basis of the full proteome profile [65], [74]. Data was imported into SIMCA 9.0 software (Umetrics, Umea, Sweden) as previously described [38]. Only protein spots present in at least 11 out of 14 (>75%) images were included in the analyses [65], [74].

### Western Blot Validation

Brain tissues from CPu (n = 8) and PFC (n = 6) of equal numbers of male and female saline and PolyI:C-treated mice were homogenized at 4°C in lysis buffer (1∶5, wt/vol) containing 1 mM EDTA and 20 mM phenylmethylsulphonyl fluoride. The proteins from resultant supernatant were determined (Bio-Rad Protein Assay) for Sodium Dodecyl-sulphate Polyacrylamide gel electrophoresis (SDS-PAGE). Proteins (20 µg/lane) were subjected to electrophoresis on a 12% (wt/vol) polyacrylamide gel in SDS, and gels subsequently processed for electroblotting to polyvinylidene difluoride (PVDF) membranes. The blotted PVDF membranes were saturated with 5% (wt/vol) of skimmed milk in Tris Buffer Saline, pH 7.4 and 0.1% (vol/vol) of Tween 20 for 1 h at room temperature. The membranes were sequentially incubated with primary antibodies to the following proteins: HSP90 (rabbit polyclonal IgG antibody, 1∶2000 dilution, Cat # ab13495, Abcam, MA, USA), HSP60 (rabbit polyclonal IgG antibody, 1∶2000 dilution, Cat # ab68416, Abcam, MA, USA), α-enolase (rabbit polyclonal IgG antibody, 1∶5000 dilution, Cat # ab49343, Abcam, MA, USA), 14-3-3 zeta (rabbit polyclonal IgG antibody, 1∶1000 dilution, Cat # ab51129, Abcam, MA, USA), eIF4A2 (rabbit polyclonal IgG antibody, 1∶1000 dilution, Cat # ab31218, Abcam, MA, USA), MEK1 (rabbit polyclonal IgG antibody, 1∶500 dilution, Cat # ab59294, Abcam, MA, USA), ERK (rabbit polyclonal IgG antibody, 1∶1000 dilution, Cat # sc94, Santa Cruz Biotechnology, CA, USA) and pERK (mouse monoclonal IgG antibody, 1∶500 dilution, Cat # sc7383, Santa Cruz Biotechnology, CA, USA), overnight at 4°C following by a peroxidase-labeled anti-mouse/rabbit IgG (1∶2000 dilution, Boehringer Mannheim, Germany) for 1 h at room temperature. After thorough washing, the positive bands were revealed using ECL western blotting detection reagents and autoradiography film (Amersham, Biosciences, UK). The intensities of the bands were quantified using IMAGE QUANT software (Molecular Dynamics, USA).

## References

[1] Chua SE, Cheung C, Cheung V, Tsang JT, Chen EY (2007). Cerebral grey, white matter and csf in never-medicated, first-episode schizophrenia.. Schizophr Res.

[2] McAlonan GM, Cheung V, Cheung C, Suckling J, Lam GY (2005). Mapping the brain in autism. A voxel-based MRI study of volumetric differences and intercorrelations in autism.. Brain.

[3] Alexander GE, DeLong MR, Strick PL (1986). Parallel organization of functionally segregated circuits linking basal ganglia and cortex.. Annu Rev Neurosci.

[4] Miller EK, Cohen JD (2001). An integrative theory of prefrontal cortex function.. Annu Rev Neurosci.

[5] Weinberger DR, Berman KF, Zec RF (1986). Physiologic dysfunction of dorsolateral prefrontal cortex in schizophrenia. I. Regional cerebral blood flow evidence.. Arch Gen Psychiatry.

[6] McAlonan GM, Daly E, Kumari V, Critchley HD, van Amelsvoort T (2002). Brain anatomy and sensorimotor gating in Asperger's syndrome.. Brain.

[7] Cheung C, Chua SE, Cheung V, Khong PL, Tai KS (2009). White matter fractional anisotrophy differences and correlates of diagnostic symptoms in autism.. J Child Psychol Psychiatry.

[8] Cheung V, Cheung C, McAlonan GM, Deng Y, Wong JG (2008). A diffusion tensor imaging study of structural dysconnectivity in never-medicated, first-episode schizophrenia.. Psychol Med.

[9] Leung M, Cheung C, Yu K, Yip B, Sham P (2009). Gray Matter in First-Episode Schizophrenia Before and After Antipsychotic Drug Treatment..

[10] Hazlett EA, Buchsbaum MS, Zhang J, Newmark RE, Glanton CF (2008). Frontal-striatal-thalamic mediodorsal nucleus dysfunction in schizophrenia-spectrum patients during sensorimotor gating.. Neuroimage.

[11] Just MA, Cherkassky VL, Keller TA, Kana RK, Minshew NJ (2007). Functional and anatomical cortical underconnectivity in autism: evidence from an FMRI study of an executive function task and corpus callosum morphometry.. Cereb Cortex.

[12] Kumari V, Gray JA, Geyer MA, ffytche D, Soni W (2003). Neural correlates of tactile prepulse inhibition: a functional MRI study in normal and schizophrenic subjects.. Psychiatry Res.

[13] Tannu NS, Hemby SE (2006). Two-dimensional fluorescence difference gel electrophoresis for comparative proteomics profiling.. Nat Protoc.

[14] Unlu M, Morgan ME, Minden JS (1997). Difference gel electrophoresis: a single gel method for detecting changes in protein extracts.. Electrophoresis.

[15] Behan A, Byrne C, Dunn MJ, Cagney G, Cotter DR (2008). Proteomic analysis of membrane microdomain-associated proteins in the dorsolateral prefrontal cortex in schizophrenia and bipolar disorder reveals alterations in LAMP, STXBP1 and BASP1 protein expression.. Mol Psychiatry.

[16] Clark D, Dedova I, Cordwell S, Matsumoto I (2006). A proteome analysis of the anterior cingulate cortex gray matter in schizophrenia.. Mol Psychiatry.

[17] Nesvaderani M, Matsumoto I, Sivagnanasundaram S (2009). Anterior hippocampus in schizophrenia pathogenesis: molecular evidence from a proteome study.. Aust N Z J Psychiatry.

[18] Novikova SI, He F, Cutrufello NJ, Lidow MS (2006). Identification of protein biomarkers for schizophrenia and bipolar disorder in the postmortem prefrontal cortex using SELDI-TOF-MS ProteinChip profiling combined with MALDI-TOF-PSD-MS analysis.. Neurobiol Dis.

[19] Prabakaran S, Swatton JE, Ryan MM, Huffaker SJ, Huang JT (2004). Mitochondrial dysfunction in schizophrenia: evidence for compromised brain metabolism and oxidative stress.. Mol Psychiatry.

[20] Iwazaki T, McGregor IS, Matsumoto I (2006). Protein expression profile in the striatum of acute methamphetamine-treated rats.. Brain Res.

[21] Iwazaki T, McGregor IS, Matsumoto I (2007). Protein expression profile in the striatum of rats with methamphetamine-induced behavioral sensitization.. Proteomics.

[22] Vercauteren FG, Flores G, Ma W, Chabot JG, Geenen L (2007). An organelle proteomic method to study neurotransmission-related proteins, applied to a neurodevelopmental model of schizophrenia.. Proteomics.

[23] Brown AS, Susser ES (2002). In utero infection and adult schizophrenia.. Ment Retard Dev Disabil Res Rev.

[24] Chess S, Fernandez P, Korn S (1978). Behavioral consequences of congenital rubella.. J Pediatr.

[25] Sham PC, O'Callaghan E, Takei N, Murray GK, Hare EH (1992). Schizophrenia following pre-natal exposure to influenza epidemics between 1939 and 1960.. Br J Psychiatry.

[26] Takei N, Mortensen PB, Klaening U, Murray RM, Sham PC (1996). Relationship between in utero exposure to influenza epidemics and risk of schizophrenia in Denmark.. Biol Psychiatry.

[27] Li Q, Cheung C, Wei R, Cheung V, Hui ES (2010). Voxel-based analysis of postnatal white matter microstructure in mice exposed to immune challenge in early or late pregnancy.. Neuroimage.

[28] Li Q, Cheung C, Wei R, Hui ES, Feldon J (2009). Prenatal immune challenge is an environmental risk factor for brain and behavior change relevant to schizophrenia: evidence from MRI in a mouse model.. PLoS ONE.

[29] Shi L, Fatemi SH, Sidwell RW, Patterson PH (2003). Maternal influenza infection causes marked behavioral and pharmacological changes in the offspring.. J Neurosci.

[30] Winter C, Djodari-Irani A, Sohr R, Morgenstern R, Feldon J (2009). Prenatal immune activation leads to multiple changes in basal neurotransmitter levels in the adult brain: implications for brain disorders of neurodevelopmental origin such as schizophrenia.. Int J Neuropsychopharmacol.

[31] Meyer U, Feldon J, Schedlowski M, Yee BK (2006). Immunological stress at the maternal-foetal interface: a link between neurodevelopment and adult psychopathology.. Brain Behav Immun.

[32] Fatemi SH, Pearce DA, Brooks AI, Sidwell RW (2005). Prenatal viral infection in mouse causes differential expression of genes in brains of mouse progeny: a potential animal model for schizophrenia and autism.. Synapse.

[33] Meyer U, Feldon J (2010). Epidemiology-driven neurodevelopmental animal models of schizophrenia.. Prog Neurobiol.

[34] Meyer U, Feldon J, Fatemi SH (2009). In-vivo rodent models for the experimental investigation of prenatal immune activation effects in neurodevelopmental brain disorders.. Neurosci Biobehav Rev.

[35] Wang Y, Lam KS, Lam JB, Lam MC, Leung PT (2007). Overexpression of angiopoietin-like protein 4 alters mitochondria activities and modulates methionine metabolic cycle in the liver tissues of db/db diabetic mice.. Mol Endocrinol.

[36] Smith SEP, Patterson PH, Siegel A, Zalcman SS (2008). Alteration of Neurodevelopment and behavior by Maternal Immune ActivationAlteration of Neurodevelopment and behavior by Maternal Immune ActivationAlteration of Neurodevelopment and Behavior by Maternal Immune Activation.. The Neuroimmunological Basis of Behavior and Mental Disorders: Springer US.

[37] Heinrich PC, Behrmann I, Haan S, Hermanns HM, Muller-Newen G (2003). Principles of interleukin (IL)-6-type cytokine signalling and its regulation.. Biochem J.

[38] Zhao B, Schwartz JP (1998). Involvement of cytokines in normal CNS development and neurological diseases: recent progress and perspectives.. J Neurosci Res.

[39] Marx CE, Jarskog LF, Lauder JM, Lieberman JA, Gilmore JH (2001). Cytokine effects on cortical neuron MAP-2 immunoreactivity: implications for schizophrenia.. Biol Psychiatry.

[40] Hans A, Bajramovic JJ, Syan S, Perret E, Dunia I (2004). Persistent, noncytolytic infection of neurons by Borna disease virus interferes with ERK 1/2 signaling and abrogates BDNF-induced synaptogenesis.. FASEB J.

[41] Kyosseva SV, Elbein AD, Griffin WS, Mrak RE, Lyon M (1999). Mitogen-activated protein kinases in schizophrenia.. Biol Psychiatry.

[42] Arion D, Unger T, Lewis DA, Levitt P, Mirnics K (2007). Molecular evidence for increased expression of genes related to immune and chaperone function in the prefrontal cortex in schizophrenia.. Biol Psychiatry.

[43] Fumagalli F, Frasca A, Sparta M, Drago F, Racagni G (2006). Long-term exposure to the atypical antipsychotic olanzapine differently up-regulates extracellular signal-regulated kinases 1 and 2 phosphorylation in subcellular compartments of rat prefrontal cortex.. Mol Pharmacol.

[44] Mill J, Tang T, Kaminsky Z, Khare T, Yazdanpanah S (2008). Epigenomic profiling reveals DNA-methylation changes associated with major psychosis.. Am J Hum Genet.

[45] Berry-Kravis E, Potanos K (2004). Psychopharmacology in fragile X syndrome–present and future.. Ment Retard Dev Disabil Res Rev.

[46] Sengupta TK, Talbot ES, Scherle PA, Ivashkiv LB (1998). Rapid inhibition of interleukin-6 signaling and Stat3 activation mediated by mitogen-activated protein kinases.. Proc Natl Acad Sci U S A.

[47] Kolch W (2000). Meaningful relationships: the regulation of the Ras/Raf/MEK/ERK pathway by protein interactions.. Biochem J.

[48] Berg D, Holzmann C, Riess O (2003). 14-3-3 proteins in the nervous system.. Nat Rev Neurosci.

[49] Pearl LH (2005). Hsp90 and Cdc37 – a chaperone cancer conspiracy.. Curr Opin Genet Dev.

[50] Stephanou A, Isenberg DA, Akira S, Kishimoto T, Latchman DS (1998). The nuclear factor interleukin-6 (NF-IL6) and signal transducer and activator of transcription-3 (STAT-3) signalling pathways co-operate to mediate the activation of the hsp90beta gene by interleukin-6 but have opposite effects on its inducibility by heat shock.. Biochem J.

[51] Kim JJ, Lee SJ, Toh KY, Lee CU, Lee C (2001). Identification of antibodies to heat shock proteins 90 kDa and 70 kDa in patients with schizophrenia.. Schizophr Res.

[52] Roncada P, Bortolato M, Frau R, Saba P, Flore G (2009). Gating deficits in isolation-reared rats are correlated with alterations in protein expression in nucleus accumbens.. J Neurochem.

[53] Corfas G, Roy K, Buxbaum JD (2004). Neuregulin 1-erbB signaling and the molecular/cellular basis of schizophrenia.. Nat Neurosci.

[54] Bronchud MH (2008). Principles of molecular oncology.

[55] Butler MG, Dasouki MJ, Zhou XP, Talebizadeh Z, Brown M (2005). Subset of individuals with autism spectrum disorders and extreme macrocephaly associated with germline PTEN tumour suppressor gene mutations.. J Med Genet.

[56] Varga EA, Pastore M, Prior T, Herman GE, McBride KL (2009). The prevalence of PTEN mutations in a clinical pediatric cohort with autism spectrum disorders, developmental delay, and macrocephaly.. Genet Med.

[57] Neves-Pereira M, Muller B, Massie D, Williams JH, O'Brien PC (2009). Deregulation of EIF4E: a novel mechanism for autism.. J Med Genet.

[58] Moy SS, Nadler JJ (2008). Advances in behavioral genetics: mouse models of autism.. Mol Psychiatry.

[59] Steelman LS, Abrams SL, Whelan J, Bertrand FE, Ludwig DE (2008). Contributions of the Raf/MEK/ERK, PI3K/PTEN/Akt/mTOR and Jak/STAT pathways to leukemia.. Leukemia.

[60] To MD, Perez-Losada J, Mao JH, Balmain A (2005). Crosstalk between Pten and Ras signaling pathways in tumor development.. Cell Cycle.

[61] Min WW, Yuskaitis CJ, Yan Q, Sikorski C, Chen S (2009). Elevated glycogen synthase kinase-3 activity in Fragile X mice: key metabolic regulator with evidence for treatment potential.. Neuropharmacology.

[62] Agarwal-Mawal A, Qureshi HY, Cafferty PW, Yuan Z, Han D (2003). 14-3-3 connects glycogen synthase kinase-3 beta to tau within a brain microtubule-associated tau phosphorylation complex.. J Biol Chem.

[63] Yuskaitis CJ, Mines MA, King MK, Sweatt JD, Miller CA (2010). Lithium ameliorates altered glycogen synthase kinase-3 and behavior in a mouse model of fragile X syndrome.. Biochem Pharmacol.

[64] Paulson L, Martin P, Nilsson CL, Ljung E, Westman-Brinkmalm A (2004). Comparative proteome analysis of thalamus in MK-801-treated rats.. Proteomics.

[65] Prabakaran S, Wengenroth M, Lockstone HE, Lilley K, Leweke FM (2007). 2-D DIGE analysis of liver and red blood cells provides further evidence for oxidative stress in schizophrenia.. J Proteome Res.

[66] Wang XF, Wang D, Zhu W, Delrahim KK, Dolnak D (2003). Studies characterizing 60 kda autoantibodies in subjects with schizophrenia.. Biol Psychiatry.

[67] Terrier B, Degand N, Guilpain P, Servettaz A, Guillevin L (2007). Alpha-enolase: a target of antibodies in infectious and autoimmune diseases.. Autoimmun Rev.

[68] Muller N, Schwarz M (2006). Schizophrenia as an inflammation-mediated dysbalance of glutamatergic neurotransmission.. Neurotox Res.

[69] Pardo CA, Vargas DL, Zimmerman AW (2005). Immunity, neuroglia and neuroinflammation in autism.. Int Rev Psychiatry.

[70] Martins-de-Souza D, Gattaz WF, Schmitt A, Rewerts C, Maccarrone G (2009). Prefrontal cortex shotgun proteome analysis reveals altered calcium homeostasis and immune system imbalance in schizophrenia.. Eur Arch Psychiatry Clin Neurosci.

[71] Fountoulakis M, Tsangaris GT, Maris A, Lubec G (2005). The rat brain hippocampus proteome.. J Chromatogr B Analyt Technol Biomed Life Sci.

[72] Suginta W, Karoulias N, Aitken A, Ashley RH (2001). Chloride intracellular channel protein CLIC4 (p64H1) binds directly to brain dynamin I in a complex containing actin, tubulin and 14-3-3 isoforms.. Biochem J.

[73] Alban A, David SO, Bjorkesten L, Andersson C, Sloge E (2003). A novel experimental design for comparative two-dimensional gel analysis: two-dimensional difference gel electrophoresis incorporating a pooled internal standard.. Proteomics.

[74] Karp NA, Griffin JL, Lilley KS (2005). Application of partial least squares discriminant analysis to two-dimensional difference gel studies in expression proteomics.. Proteomics.

